# Challenges to Students’ Learning and Wellbeing During Placement Abroad: A Qualitative Study Using Rich Pictures

**DOI:** 10.5334/pme.1618

**Published:** 2024-12-26

**Authors:** Miriam H. Wijbenga, Wieke E. van der Goot, Stephan P. J. Ramaekers, Pim W. Teunissen, Robbert J. Duvivier, Erik W. Driessen

**Affiliations:** 1 European School of Physiotherapy within the Faculty of Health, Amsterdam University of Applied Sciences, Amsterdam, The Netherlands; 2 School of Health Professions Education (SHE), Faculty of Health, Medicine and Life sciences, Maastricht University, Maastricht, The Netherlands; 3 Postgraduate medical education, Martini Academy, Martini Hospital Groningen, The Netherlands; 4 Wenckebach Institute for Education and Training (WIOO), Lifelong Learning, Education and Assessment Research Network (LEARN), University of Groningen, University Medical Center Groningen, Groningen, The Netherlands; 5 Center of Expertise Urban Vitality, Faculty of Health, Amsterdam University of Applied Sciences, Amsterdam, The Netherlands; 6 Maastricht University Medical Center (MUMC+), Maastricht, The Netherlands; 7 Lifelong Learning, Education, and Assessment Research Network (LEARN), University Medical Center Groningen, The Netherlands; 8 School of Medicine and Public Health, University of Newcastle in Australia, Australia; 9 Parnassia Psychiatric Institute, The Hague, The Netherlands

## Abstract

**Introduction:**

Undergraduate healthcare students on placement abroad can experience challenges that affect their wellbeing, personal and professional development. These challenges may result in students taking a more peripheral role in workplace activities, which negatively impacts learning. We studied *how personal and professional challenges affect students’ learning and wellbeing during a clinical placement abroad*.

**Methods:**

We used the rich pictures drawing method to elicit semi-structured student interviews and capture personal and professional challenges within different contexts. Language, culture, education, and belonging were used as sensitizing concepts, underlying thematic analysis. We conducted a parallel and iterative analysis of the transcripts and rich pictures. Team discussions focused on developing patterns and further conceptualization of results.

**Results:**

Based on thirteen student accounts, we identified four main themes: ‘Learning to work in the international context’; ‘Cultural differences shape professional identity’; ‘Deliberate social connections’; and ‘Personal growth through international experiences’. Active participation in local practices was crucial to overcome barriers in language, culture or education, and increase belonging. Local healthcare teams and peers supported students’ wellbeing, personal and professional development by helping them establish their role as a learner, whilst exploring the scope and boundaries of their future profession.

**Conclusions:**

Language, cultural and educational challenges can be considered an inevitable part of student placement abroad. Local peers and staff may support this transition and help recognize learning opportunities and challenges in the workplace. Clinical educators can facilitate learning and wellbeing by providing social support and guidance on professional behavior, including communication.

## Introduction

Students participating in a placement abroad are faced with multiple challenges both in and outside the workplace, such as navigating a different healthcare system, familiarizing themselves with local customs, or simply managing daily tasks within an unfamiliar environment [1,2]. Although recent literature confirms the positive impact of international experiences [3,4], the complex nature of professional and personal challenges during placement abroad may also lead to feelings of isolation, exhaustion and loneliness [2]. Whereas discomfort and unexpected experiences can be considered part of the learning process [5,6,7], it is unknown how those challenges affect the students’ learning and wellbeing, especially during placement outside their country of training [3].

Considering the social nature of workplace learning, students should be seen as active stakeholders in workplace learning, instead of passive consumers of clinical education [8]. Newcomer socialization during initiation of international placement can be enhanced by knowledge regarding the organization, and expectations of those working in the setting [9]. Participation in practice may be supported by universal standards and universal equipment, such as therapeutic interventions, which helps students in their role as ‘legitimate participants’ in practice [10,11].

Through interactions with the local healthcare team, students learn to overcome differences in healthcare systems, professional practices or educational approach [12,13,14]. However, language barriers may hinder these learning interactions, for example limiting informal communication between the student and patients or staff [7]. Cultural diversity may further complicate interactions between the student, professionals, patients, and peers, based on different understandings of healthcare [15,16]. Also, different educational approaches are likely to contribute to the workplace-related challenges students perceive, adding up to their overall sense of belonging, depending on the individual and the context [2,17]. A recent meta-ethnography study describes how international medical graduates (IMG) can perceive four different barriers in language, culture, education and belonging, depending on the unique degree of dissonance between the IMG and the host country [17]. For this study, we will use these four barriers as sensitizing concepts to identify the challenges students face while on placement abroad.

To better understand the complex nature and diversity of the challenges related to workplace learning outside the students’ country of training, we studied the following research question: *How do personal and professional challenges affect student learning and wellbeing during a clinical placement abroad?*

## Methods

### Design

In this qualitative study, we adopted a constructivist thematic analysis to capture the complex nature of workplace learning in international contexts [8,18,19]. We used rich pictures, which are a visual representation created by a research participant of a particular situation, to elicit students’ perceived challenges [20]. The rich pictures methodology is suitable for studying complex and dynamic phenomena and allows participants to visualize their experience and some of its complexity, including context, people, relations, symbols, metaphors, and emotions, and therefore may enrich recollection of memory [21,22,23]. As detailed below, we conducted our study within physiotherapy, which provides a relevant field to study the personal, professional and contextual challenges encountered by students, as it involves multiple placements in different healthcare settings, ranging from small private clinics to large, multidisciplinary, teaching hospitals.

### Setting and participants

Participants were recruited from a three-year, English-taught, international programme (BSc. Hons) at the European School of Physiotherapy (ESP), Amsterdam University of Applied Sciences, the Netherlands. Students complete 10-week clinical placements, in a variety of healthcare settings, where they work towards independent practice under the supervision of professional therapists. Placements can be self-organised or obtained through the school’s database. Student preparation includes practical matters and individual learning goals. An academic mentor is available online during the placement period to monitor progress. Minimally two out of four placements take place outside the country of training, for students to experience different perspectives on healthcare and professional approaches. We drew a purposive sample among all Year 1 and 2 students who completed placement abroad between June – August 2023 (n = 37/72, 52% of the placement cohort). Students who completed a placement in the Netherlands were excluded from participation in this study. Participants received an information letter via email, followed by a personal invitation two weeks later. Eleven students volunteered to participate in September 2023. Four of them completed placement in their home country. To enrich the developed themes, we included Year 3 students who completed placement abroad for interviews in January 2024 (n = 2/7, 29% of the placement cohort). Participants (Table 1) signed informed consent before data collection began and consented to publication of their drawings. Given the voluntary nature of the research, participants were able to withdraw at any time.

**Table 1:** Demographics.


	PARTICIPANT CHARACTERISTICS (N)

*Gender*	Female (n = 6); Male (n = 7)

*Age*	27 years (mean); 19–40 years (range)

*Nationality*	European (n = 9); non-European (n = 4)

*Region of placement*	Europe (n = 8); North America (n = 2); Africa (n = 3)

*Main language at placement*	Dutch/Flemish (n = 4), English (n = 6), other (n = 3)


### Data collection

MHW organised individual face-to-face meetings with all participants. Participants were instructed to draw a rich picture of their most memorable moment [24], related to individual challenges during placement abroad. After approximately 30 minutes, MHW interviewed the participant about the story behind their drawing before continuing to explore its different elements together to get a full understanding of the rich picture (Appendix B). Finally, the interview focused on the degree of dissonance, specifically aimed at language, culture, physiotherapy education and belonging, in relation to student learning and wellbeing during placement abroad. All interviews were audio recorded in English, lasting between 30–45 minutes.

### Data analyis

Interview recordings were transcribed verbatim and pseudonymized, after which MHW sent each participant an individual copy to check for agreement, before further analysis. MHW and WEvdG familiarized themselves with the data by individually coding the first two transcripts, following a thematic analysis approach [18]. Sensitizing concepts were applied as a priori themes to deductively code the dataset, while also inductively identifying new codes from the verbatim transcripts. Original drawings were collected and stored separately by MHW directly after the interview, without any identifying information. Once the first interview round was done, eleven drawings were shared with the research team (WEvdG, SPJR, PWT, RJD, EWD). After exploring the use of space, colour and different elements, the team discussed their general interpretations of each drawing. Next, MHW shared the story behind the drawings, to further discuss the meaning and assumptions behind them and connect the drawings with the interviews. Finally, all drawings were presented simultaneously, to further look for similarities and differences, and identify patterns in the drawings, whilst reflecting on the sensitizing concepts [17]. MHW guided the gallery walk and audio recorded the discussions [24]. The rich picture analysis provided direction for further coding of interview data.

MHW, WEvdG and RJD coded and discussed three more interview transcripts, using team discussions to categorize the data into themes, before MHW coded another six transcripts by hand. Last, MHW conducted three more interviews, focused on contrasting or new insights to enrich the developed themes. The final interview was rejected for analysis, because the participant obtained working experience at the clinic prior to placement. For reasons of reflexivity and transparency, MHW has kept an audit trail, including notes on team meetings, decisions, reflexivity and memos [25].

### Reflexivity

Data interpretation and analysis was done from a constructivist perspective. All team members have been involved in international collaboration or exchange projects, either clinically or via research. Their experiences as (allied) healthcare professionals (MHW, SPJR, PWT, RJD) and educationalists (MHW, WEvdG, SPJR, PWT, EWD) shaped their interpretations of the drawings and transcripts. MWH, SPJR, PWT and RJD have experienced placements abroad as students and professionals, which has helped them to contextualize the data. EWD studies international healthcare and has worked in international contexts. All authors have worked in, designed and/or researched workplace learning in different fields of Health Professions Education (HPE). MHW is directly involved in the ESP programme as a trained physiotherapist, lecturer, and clinical coordinator. Her international experiences and close connection to the students facilitated the understanding of challenges during placement abroad. WEvdG conducts research on motivation and well-being in HPE. Her previous experience with rich pictures [23] made her sensitive to experiences of motivation and belonging related to workplace learning. SPJR’s specific focus concerned the way professional reasoning and (interprofessional) team learning is affected by international settings. PWT’s experience as a HPE professional and researcher has sensitized him to focus on workplace learning, transitions and learner well-being. RJD holds a broad interest in workplace learning and focused on the role of supervisors in helping students participate. EWD’s background made him sensitive to participants’ experiences with power issues and tensions in the workplace, and how participant experiences related to workplace-based learning theories. The team shares a critical stance towards short-term placements and their ability to offer meaningful learning experiences.

## Results

Based on the rich pictures and interview data, we have identified many personal and professional challenges that students face while on placement abroad. We developed four main themes underlying student learning and wellbeing: Learning to work in the international context; Cultural differences shape professional identity; Deliberate social connections; and Personal growth through international experiences. Within these themes, we will describe how challenges in language, culture, education and belonging affected student learning and wellbeing, illustrated with participants’ quotes and drawings.

### Learning to work in the international context

Although clinical teaching and assessment methods often differed from their educational context, most students seemed to adapt to different professional approaches, including cultural aspects related to the healthcare system and provision of care. Learning conversations and practical assistance from local team members supported students’ understanding of different approaches to healthcare, including learning how to use unfamiliar professional equipment, such as electrotherapy devices. The opportunity to use familiar equipment and spend time on administration or team activities helped students adapt to their new environment. Additionally, the presence of local peers enabled students to share workplace experiences and practice their skills in a more informal way, which greatly enhanced their sense of belonging to the community of practice: ‘I think I have also opened up a bit more to the patients once [my peers] were present in the hospital […] taking off workload. You know, they would introduce me to other stuff. We would discuss, talk, and yeah, it was much better to have colleagues than being alone.’ (P5) Whereas communication with local healthcare teams was often in English, and English was also the main language during more formal learning conversations, almost all students encountered language barriers. Different nuances between languages would add to the effort students had to make to gather and process clinical information: ‘[…] sometimes, in a conversation, I would be staring at [my clinical instructor], you know, to try and grasp- like: Okay, I got it. It was a lot of effort to stay focused.’ (P6) Additionally, language differences would hinder patient communication, for example when students were confronted with regional dialects or local languages such as Flemish (Belgium) or Xhosa (South Africa). Despite the difficulties communicating in another language, most students felt able to engage with their new learning environment straight away. Students appreciated invitations to participate, actively looking for opportunities to boost their self-confidence and help establish their position as a learner within the workplace: ‘Sometimes I go in and ask: “OK, maybe we can do something differently?” or: “Can I advise something?” Or they ask me something. That really worked. I liked that.’ (P9) Furthermore, students described how workplace affordances facilitated a quick integration into the local team: ‘I think because I was the only physiotherapy intern that after a few weeks people knew my face, like they knew “Oh the patient is going for physiotherapy, because you are here.”’ (P3)

### Cultural differences shape professional identity

**Figure 1:**
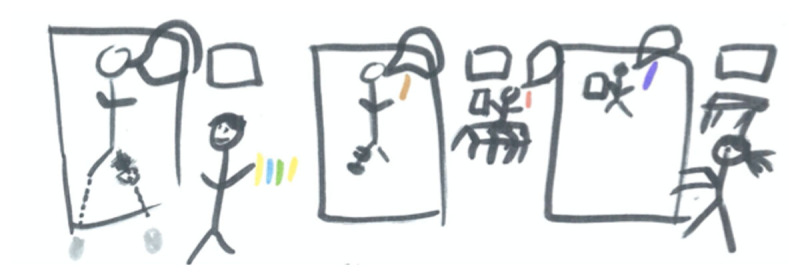
Communication tools. *Note*: The coloured stripes in this picture represent coloured pencils, which the student (P5) brought to the pediatric ward during treatment hours, together with a whiteboard: ‘I would use the board to like explain how, like, what happened to their bodies and how they experience change, and what pain is.’ Besides using this for educational purposes, the student would hand out pencils and colour books to keep the children busy, while attending to others. To receive a turn, the children had to call on the student, evoking frequent interactions, despite the language barrier.

Experiencing different healthcare systems and having an opportunity to compare professional and educational approaches in another context made students reflect on their future role as healthcare professionals: ‘You realize how, even after you can get your degree, you can do whatever, but it still takes a certain person to be a physio, or any healthcare practitioner.’ (P4) Yet, different cultural interpretations, such as with non-verbal communication across nationalities, represented additional challenges to patient-related tasks. To overcome initial language barriers and enable full participation in practice, students switched to non-verbal communication or used communication tools (Figure 1). This creative approach allowed the students to strengthen their role as caregivers and increased their sense of belonging within the local community of practice.

**Figure 2:**
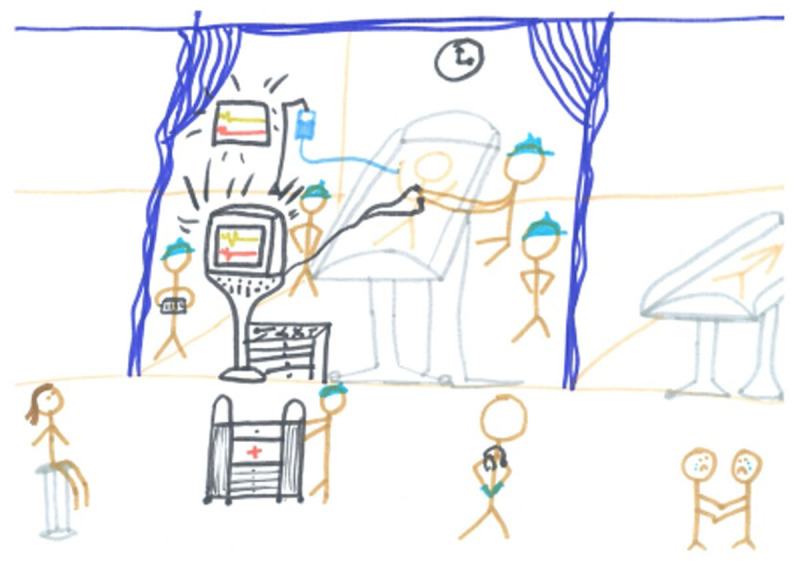
Boundaries and belonging in the clinical environment. *Note*. Unexpected situations provided valuable learning opportunities and enhanced students’ sense of belonging. A student (P4, left) witnessed the resuscitation of a patient, whilst waiting for her supervisor to return. The attending staff worked together as a ‘well-oiled machine’: ‘Everyone was so calm. Someone was handing the tools; another was taking notes. They were watching the time to switch over. [Someone] was pushing the cart. It was all just controlled chaos.’ The observation, though traumatic in nature, made the student reflect on her own role and position in the clinic: ‘In reality, you’re helpless in these situations. I mean, they do what they can. […] To some extent, I felt like I was invading [the patient’s] privacy. But at the same time, I reminded myself that this is going to, if I work in a hospital, this is going to be the reality of things.’ The response of the healthcare team and individual support received after losing the patient made this experience an unforgettable lesson.

For some students, differences in workplace culture and organization made it hard to meet expectations: ‘They think that my- the way I see how physiotherapy should be working and how they do their business is different.’ (P9), whilst generally students quickly learned how to professionally relate to their new environment through social interactions with peers and other healthcare professionals. Often, placement abroad provided students with a wider range of practical experiences, including complex care, such as ICU. This presented unexpected challenges, such as overcoming the loss of a patient whilst having to continue clinical work (Figure 2). To learn how to cope, students needed the support of colleagues and peers: ‘You’re not in control of the situation, even though you have control of the thirty minutes that you treat your patient for. With our work, you’re not in control of anything in reality.’ (P4) Overall, students indicated that collaborative learning was valuable to their placement experience, since it increased their focus on content and learning opportunities, besides teaching them how to be adaptive and establish a professional balance between personal and work-related challenges.

### Deliberate social connections

Students felt that engagement with peers, family and friends was crucial to make sense of their individual experiences abroad. Social support, whether close by or more distant, proved indispensable to cope with personal challenges like stress, financial support, or loss of motivation. Conditional factors for placement, such as travel distance, accommodation or financial support would also challenge individuals, trying to find a sense of belonging: ‘I was staying in a very small room that I had to share with someone else. And sometimes it felt a little bit suffocating, which is OK because all I was doing was studying and revising.’ (P13) Some students deliberately used their physical distance to friends and family for temporary self-isolation and to confront personal dilemmas, whilst placement offered structure, distraction and social interaction during the daytime. Others expressed how the challenge of balancing work, study and personal wellbeing left little room to engage in social interactions in the workplace: ‘No one would ask: “How are you today?” It might have been more of a cultural thing, but people not asking those questions was sometimes quite nice because often I’d had a rough weekend.’ (P13). Often, students made a deliberate choice about where to invest, given the short nature of placements.

### Personal growth through international experiences

**Figure 3:**
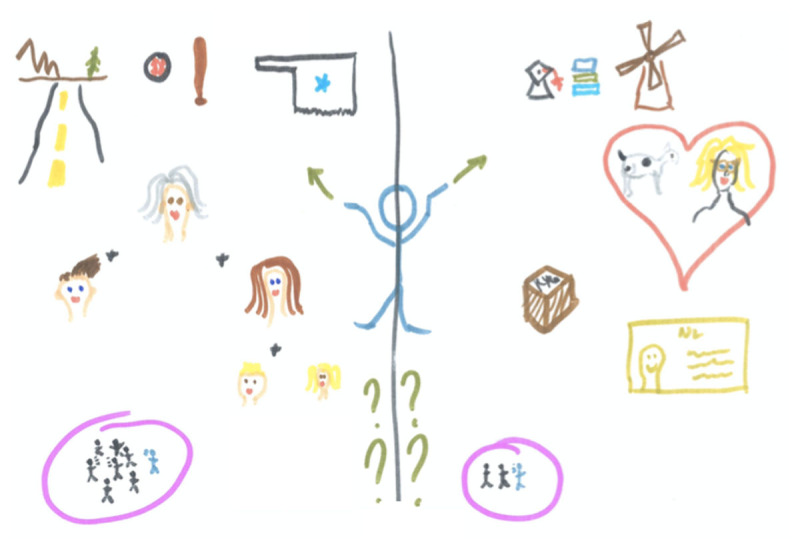
Questioning belonging. *Note*. One participant (P11) felt torn between his home country and the Netherlands, where he currently resides: ‘… you start working there and- I don’t know, suddenly, all the cultural things, the language, the knowing, the area, all that stuff started becoming a benefit to me. […] I was surprised at the outcome. I really thought after about 6 weeks I would have been like “Oh my God, I cannot wait to get out of here” and it was the exact opposite.’ The picture clearly shows his divide between culture, roots and family (on the left) versus study, work and love (on the right): ‘I have two equally important, competing, you know, units? And I don’t know where I belong. And this is a new feeling for me.’

The interviews illustrated how personal and professional challenges to learning and wellbeing tied closely into students’ engagement during placement. Many students felt their participatory experiences helped reframe and reduce the impact of personal challenges faced, thus enhancing their wellbeing and providing them with room to learn: ‘I think the biggest thing that I learned during the internship, apart from the skills that I gained, was that everybody has a struggle, but you can decide to […] handle it in a different way.’ (P2) By welcoming the students and allowing them to participate, host institutions would help the students to break down perceived barriers in professional communication and create a sense of belonging: ‘What I enjoyed most about working there is that it was so diverse and what kind of nationalities came into the clinic, because it was really interesting talking about their perspective.’ (P10) Apart from broadening their scope of practice, new perspectives on students’ personal and professional contexts sometimes triggered existential questions about belonging (Figure 3). Thus, being embedded in an international context, both educationally as well as professionally, challenged students not only to develop on a professional level, but also taught them to self-reflect and grow as a person.

## Discussion

This study investigated how personal and professional challenges affect students’ learning and wellbeing during a clinical placement abroad. Both the participants’ drawings and interviews showed that learning and wellbeing are inextricably entangled, influenced by factors in and outside the workplace. Participation in patient-related activities was crucial for students to become engaged in local physiotherapeutic practice and learn how to overcome initial language barriers. Sharing different cultural and educational perspectives on healthcare provision with local peers and healthcare professionals helped the students navigate difficulties when adjusting to the international workplace setting and to shape their professional identity. Students’ wellbeing depended on how they were able to balance personal experiences of living and working abroad with professional challenges encountered in the workplace. Deliberate use of their social support networks seemed crucial for students to deal with the personal and professional challenges involved with placement abroad.

Whereas the individual degree of dissonance will differ between international medical graduates and healthcare students on temporary placement, we have found that undergraduate students perceive similar barriers when learning to work in the international context [17]. This also applies to participants who engage in home country placements, who can be challenged by local dialects and jargon, or feel estranged from their cultural background after being educated in a different context. It seems the transition to a different clinical setting, whether this concerns a domestic placement or abroad, is mainly determined by contextual and situational factors, and healthcare students’ performance therefore depends on the local learning environment [26,27,28]. Although language barriers initially hampered patient communication, most participants were able to interact professionally, especially when feeling supported by local peers and staff [9,29,30]. By investing in relevant support networks during placement abroad, students learnt to communicate in different ways and were able to create additional opportunities for learning [31,32]. These findings resonate with previous research, showing that students who feel empowered by their learning environment can optimize individual learning strategies and reflection, in favour of personal and professional growth [28,33,34,35,36].

### Implications of findings

Students who can establish adequate support networks to find individual support when encountering personal or professional challenges during placement abroad [2,31,32], are more likely to proactively engage in local practices. Through participation, students will recognize opportunities for learning related to differences in professional, cultural or educational approach [8,12]. We described how some of these learning opportunities were triggered by professional equipment, like walking devices or therapeutic material, whereas other students relied on time spent on clinical activities or patient administration, to increase understanding of professional approaches. Our findings indicate that raised awareness to reflect on different contextual factors, including therapeutic devices and time, facilitates workplace learning [26,36]. Instead of leaving students to overcome challenges by themselves, at the risk of mental wellbeing or withdrawal from practice, the local healthcare team plays an important role in providing safe spaces for learning opportunities and self-reflection, to capitalize on individual learning experiences and professional growth [27,37]. Sheehan and Wilkinson, in their reflections on the impact of time and context in clinical learning, argue that “the best placement structure may be the one that fully utilises the unique context of the site and ensures the unique opportunities are utilised” [38]. Although some participants made a practically informed decision when choosing their placement location, instead of focusing on learning potential, we believe our findings indicate a range of additional learning opportunities in the international workplace, which could be supported in a structural way, for example by introducing peer-to-peer intervision [39]. Organising a local buddy system might ease students’ transition into the learning environment and provide them with experiences that will go beyond clinical experiences [40]. Moreover, when preparing students to go abroad, institutions should consider the goal of international placements within the undergraduate curriculum: should these be focused on content only, or include personal and professional development goals? This would help to clearly outline the role and responsibilities of existing social networks and emphasize the importance of individual strategies to successfully support students’ learning and wellbeing in an international clinical context [41,42].

### Strengths, limitations and future research

This qualitative study uses a highly diverse representation of individual student experiences, and reflects a wide variety of healthcare settings, placement conditions, educational perspectives, cultures and resources. A strength of using rich pictures as a visualization method is that students draw elements of their placement experiences that can be difficult to verbalize, thus providing additional insights by revealing contextual aspects of workplace learning abroad [22]. In addition, applying the ‘degree of dissonance’ as a sensitizing concept for data collection and analysis allowed us to zoom in on unique challenges to student learning and wellbeing, related to language, culture, education and belonging [17]. Thus, findings are constructed on students’ experiences and reflections, whereas measurement of actual changes in learning did not fall within the scope of this research. A limitation might be that the most memorable experience did not capture all daily challenges students experienced, although we explored the impact of place, time and context in our interviews. Future longitudinal research is needed to capture daily challenges and how they develop over time. Another limitation includes recruitment from a single institution, which may have influenced the degree of preparedness of these students. Nevertheless, we were able to recruit students from all years of training and they elaborated on positive and negative experiences, broadening the richness of the experiences of placements abroad. Further, our deductive analytical lens, based on a previous study of IMG’s [17] provided valuable insights that students on short placements abroad also struggle with language, cultural and educational challenges. Students’ experiences illustrated that many elements interact. However, social support may mitigate these challenges of placement abroad and facilitate professional development, learning, and wellbeing. Especially, a sense of belonging and wellbeing seems to be a shared responsibility of students, local peers and staff, as well as their social network of family and friends in their home country. The composition of the research team shaped the interpretation of the data, but our diverse backgrounds and research perspectives allowed for sharing different perspectives and experiences on placements abroad. This was facilitated by regular team briefings, a clear audit trail, constant refinement of the themes and transparent reporting. Future research involving sociocultural theories like legitimate peripheral participation may add information and facilitate healthcare students’ navigation of sociocultural differences during placement abroad and outline strategies that clinical educators may use for practical and effective support [43]. Additionally, peer-assisted learning as an educational intervention during clinical training would be an interesting area to explore, for its potential benefits to students’ learning and wellbeing, especially when going abroad.

## Conclusions

Challenges in language, culture and educational background can be considered a logical part of student placement abroad. This transition may be smoothed by local peers, who can introduce students to the educational and clinical context, to help recognize opportunities and challenges in the workplace, create learning opportunities and enhance student wellbeing. Clinical educators, therefore, should emphasize the role of existing support networks and stimulate proactive learning behaviours, including communication tools, to enhance student learning and wellbeing during placement abroad.

## Appendix A – Instructions for drawing a Rich Picture

I will begin by describing to you what the format of the interview will be. I will ask you to draw a picture. This picture will be about your most memorable experience during clinical placement abroad. We have found that during placement abroad, there can be several challenges to both personal and professional wellbeing. We are interested to discover your personal experiences as an intern in an unfamiliar healthcare setting, outside your country of training. We are also interested to find out how you were dealing with the more personal challenges during placement.

I will ask you to draw a picture. In this drawing, I invite you to represent the most memorable experience you’ve had during your time abroad as an intern. The picture you will draw is called a rich picture. A rich picture is a visual representation of a particular situation, intended to show the experience in its complexity. You are free to include any detail that you deem relevant in this picture: the people, their connections, relations, colours, objects, emotions. Important about the rich picture is the content of the drawing, not your artistic skills. You can use the different colours provided for the drawing to help picture the context and different elements of the situation. Try and use as few words as possible in your drawing. Here is an example of a published rich picture – *show example* [23, Figure 1].

You will have 30 minutes to draw your picture, during which I will be next door. I will come back in half an hour’s time, or earlier if you have finished before that. I will first ask you to tell me about the story behind the picture, before we will look at the picture together. I’ll ask you to explain what you have drawn and ask further questions to help me better understand the situation. The interview part will take approximately 30–60 minutes. I will record the conversation, so that I can analyse the interview later on. First of all: try to recall the most memorable experience of your placement abroad and picture this on your sheet.

## Appendix B – Interview Protocol

The interview will follow four phases. The first phase starts by asking the participant to share the story behind the drawing. Second, the interviewer, together with the participant, will explore the different elements of the drawing (including the use of space, colour, and symbols), looking for hidden meanings and trying to get a complete understanding of the rich picture. Third, questions like ‘What led you to choose this specific experience?’ and ‘What makes it a memorable experience?’, will lead to further clarification of the professional, personal and contextual challenges encountered. In the fourth phase the interviewer will continue to focus on the degree of dissonance, specifically aimed at language, culture, physiotherapy education and belonging, in relation to student’ learning and wellbeing during placement abroad. Finally, the interviewer will ask the participants how they experienced drawing a rich picture and if they have anything to add or would like to change anything in their drawing after having discussed the experience.
